# Infarcted Adenomatoid Tumour of Epididymis: A Rare Case Report

**DOI:** 10.1155/2013/937689

**Published:** 2013-04-03

**Authors:** A. Gupta, M. Livingston, R. Singh, D. Tansey, L. Solomon

**Affiliations:** ^1^Urology Department, Queen Alexandra Hospital, Southwick Road, Cosham, Portsmouth PO6 3LY, UK; ^2^Histopathology Department, Queen Alexandra Hospital, Southwick Road, Cosham, Portsmouth PO6 3LY, UK

## Abstract

Paratesticular tumours are pathologically rare. The vast majority are benign in nature with adenomatoid tumours representing the most common pathological entity. We present the case of a 32-year-old man, from the Indian subcontinent, who presented with a painful scrotal swelling sustained after trauma. The history suggested that the scrotal mass had been present for approximately 12 months, and a preliminary diagnosis of a haemorrhagic cyst caused by trauma was made. Initial management included scrotal support, analgesia, and a follow-up magnetic resonance imaging (MRI) scan. Subsequent imaging and then further histological analysis confirmed a partly necrotic/infarcted adenomatoid tumour of the right epididymis. After scrotal exploration and epididymectomy, the patient made a complete recovery, and, with the histological diagnosis, he was discharged with no further followup. The case is presented as a learning point in the identification and management of such pathologies.

## 1. Introduction

Paratesticular tumours are pathologically rare. The vast majority are benign in nature with adenomatoid tumours representing the most common pathological entity [1]. The predeliction of paratesticular adenomatoid tumours for men in their 3rd-4th decade of life makes them difficult to be differentiated clinically and radiologically from other more sinister intrascrotal pathology and therefore presents a diagnostic challenge [2]. We present a case that highlights this diagnostic dilemma.

## 2. Case Report

**Figure 1:**
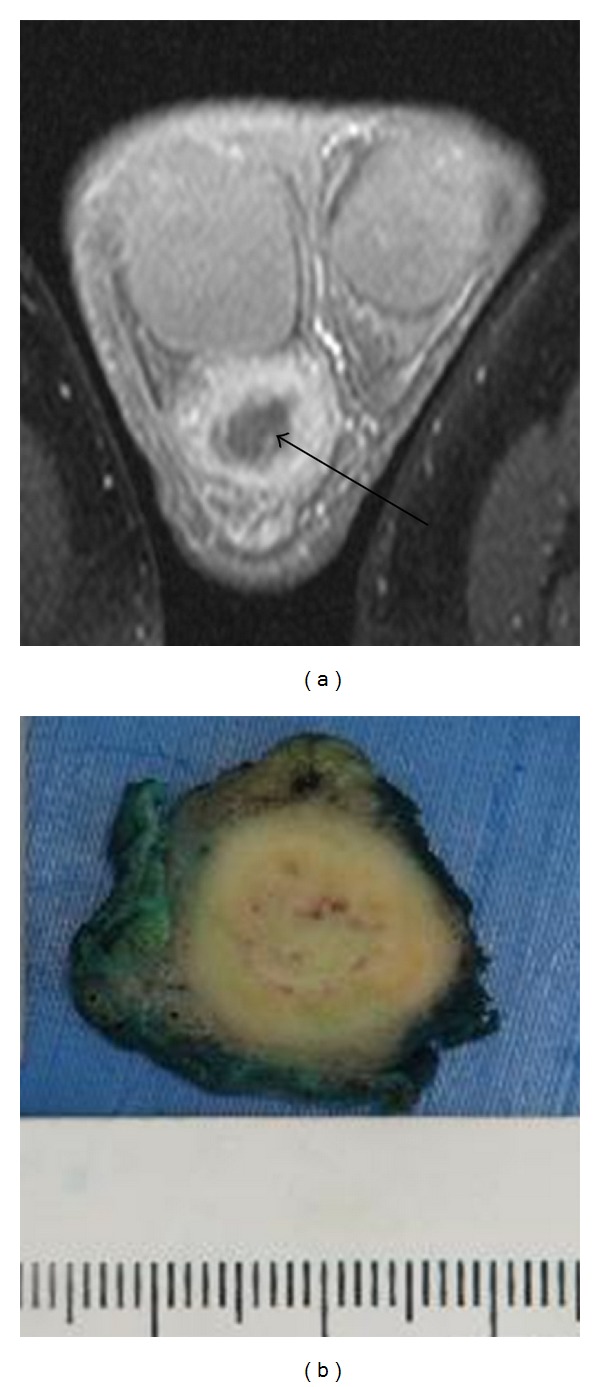
(a) Cross-sectional MRI shows peripheral enhancement of the lesion, which is clearly separate from the testis and demonstrates a central area of necrosis. (b) Cut surface of the specimen shows a yellow tumour mass with central necrosis.

A 32-year-old man form the Indian subcontinent presented to the emergency department 2 days after having been kicked in the scrotum during a football match with increasing pain and swelling of the right hemiscrotum. A firm discrete mass was noted at the lower pole of the right epididymis that was clinically inseparable from the otherwise unremarkable testicle. The right testicle was nontender and sat in a normal position with no signs of scrotal skin ecchymosis. The history suggested that the scrotal mass had been present for approximately 12 months, and a preliminary diagnosis of a haemorrhagic cyst caused by trauma was made. Initial management included scrotal support, analgesia, and a follow-up magnetic resonance imaging (MRI) scan. The MRI confirmed a 2.3 cm irregular lesion arising posterior to the lower pole of the right testis that enhanced peripherally with central necrosis and therefore could not rule out a potentially malignant lesion (Figure 1(a)). After informed consent, a scrotal exploration and epididymectomy was performed. Grossly, the specimen measured 25 mm in maximum diameter. Sectioning revealed a 20 mm yellow, partly necrotic tumour mass (Figure 1(b)).

**Figure 2:**
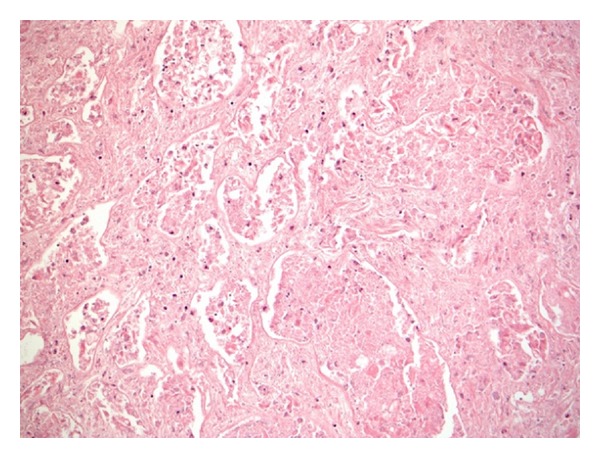
Coagulative necrosis in the central part of the tumour.

**Figure 3:**
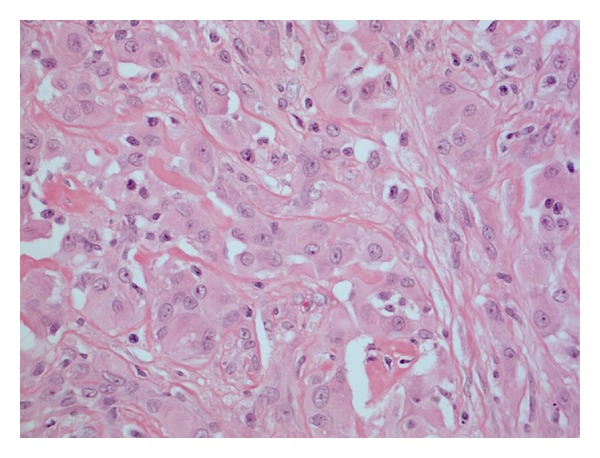
Irregular nests of viable neoplastic cells.

Histological examination (H+E) showed a central area of coagulative necrosis with surrounding reactive fibroblastic tissue and inflammation (Figure 2). Irregular clusters and solid nests of viable epithelioid cells were present, partly obscured by the fibroblastic tissue reaction (Figure 3). 

**Figure 4:**
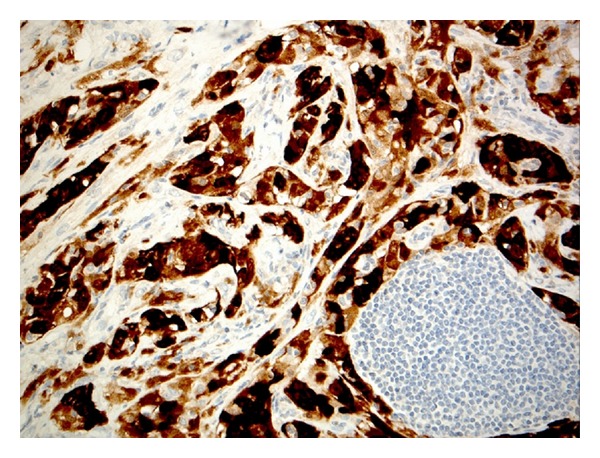
Positive calretinin staining in the neoplastic cells.

On immunohistochemical staining, these epithelioid cells were positive for CAM5.2 and CK7 and also expressed the mesothelial markers calretinin and WT1 (Figure 4). The H+E appearances and immunoprofile were consistent with a partly necrotic/infarcted adenomatoid tumour. No evidence of malignancy was seen. The tumour was well circumscribed and completely excised at all margins. The patient was informed of the results, reassured, and discharged from further followup.

## 3. Discussion

Paratesticular neoplasms are rare and account for less than 10% of all intrascrotal tumours [3]. Epididymal tumours are a rare subtype of paratesticular neoplasms with the majority being benign in nature. Adenomatoid neoplasms represent the most common histological variant followed by leiomyoma and then papillary cystadenoma with other rarer lesions including angioma, lipoma, dermoid cysts, fibroma, hamartoma, teratoma, and cholesteatoma making up the remainder. Adenomatoid tumours can be found in a variety of locations including the spermatic cord, prostate, ejaculatory ducts, and scrotal capes. In females, they can be located in the uterus, Fallopian tubes, and ovarian area [4], Only 25% of all epididymal neoplasms are found to be malignant with the most common subtype being sarcoma (45%) and the rest comprising metastatic deposits and primary epididymal carcinomas. Epididymal adenomatoid tumours are commonly present in the 3rd or 4th decade of life as a painless small lump, but they can occur at any age and can vary in size [5]. Traumatic infarction is an unusual presentation but it was first described in the literature by de Klerk and Nime in 1975 [6]. Since then, there have been a small number of case reports highlighting the diagnostic dilemma that infracted adenomatoid tumours present, but there is no consensus on how to investigate and manage them. This case represents another example of a rare presentation of this tumour. In our view, even though malignant causes are rare, there should always be a high index of suspicion when assessing and managing patients presenting with scrotal masses, and prompt multidisciplinary discussion to dictate investigations and interventions is essential.
